# CBX7 Inhibits Cell Growth and Motility and Induces Apoptosis in Cervical Cancer Cells

**DOI:** 10.1016/j.omto.2019.09.002

**Published:** 2019-09-24

**Authors:** Rong Li, Qi Yan, Ping Tian, Yan Wang, Jing Wang, Ning Tao, Li Ning, Xin Lin, Lu Ding, Jiwen Liu, Cailing Ma

**Affiliations:** 1 Postdoctoral Research Center on Clinical Medicine, First Affiliated Hospital, Xinjiang Medical University, Xinjiang, China; 2 Department of Maternal, Child and Adolescent Health, College of Public Health, Xinjiang Medical University, Xinjiang, China; 3 Postdoctoral Research Center on Public Health and Preventive Medicine, Xinjiang Medical University, Xinjiang, China; 4 Fifth Affiliated Hospital, Xinjiang Medical University, Xinjiang, China; 5 Tumor Hospital Affiliated to Xinjiang Medical University, Xinjiang, China; 6 State Key Laboratory of Pathogenesis, Prevention and Treatment of High Incidence Diseases in Central Asia (PPTHIDCA), Department of Gynecology, First Affiliated Hospital, Xinjiang Medical University, Xinjiang, China

**Keywords:** cervical cancer, CBX7, cell proliferation, invasion, apoptosis, migration, E-cadherin, motility

## Abstract

The chromobox protein homolog 7 (CBX7), one member of the polycomb group family, has been characterized mainly to play a tumor-suppressive role in human malignant neoplasias. Moreover, downregulation of CBX7 is correlated with poor prognosis and aggressiveness in a variety of human cancers. However, the biological functions and role of CBX7 in cervical cancer have not been elucidated. In the present study, we explore whether CBX7 exerts its tumor-suppressive function in cervical cancer. To achieve this goal, molecular approaches were used to upregulate the expression of CBX7 or downregulation of CBX7 in cervical cancer cell lines. We observed that overexpression of CBX7 inhibited cell growth and induced apoptosis in cervical cancer cells. CBX7 overexpression retarded cell migration and invasion in cervical cancer cells. In line with this, downregulation of CBX7 promoted cell growth and migration as well as invasion in cervical cancer cells. Our findings suggest that CBX7 might be a tumor suppressor and could be a potential target in cervical cancer.

## Introduction

Cervical cancer is one of the common leading causes of cancer death in women. There are an estimated 13,170 new cervical cancer cases and 4,250 deaths from this disease in the United States this year.^1^ Screenings in women and human papillomavirus (HPV) vaccination uptake have reduced the incidence rate of cervical cancer; however, cervical cancer is still a health problem in less-developed countries.^1^ The treatment strategies of cervical cancer include surgery, radiotherapy, and platinum-based chemotherapy.^2^ As a result of radio resistance and drug resistance, as well as metastasis, some patients with cervical cancer have poor survival rate. It is important to discover new therapeutic management to improve the treatment outcome of cervical cancer patients.

Accumulated evidence has suggested that multiple factors, including smoking, oral contraceptive use, high parity, and HPV infection, could contribute to cervical tumorigenesis.^3^ Moreover, key gene mutations, such as phosphatidylinositide 3-kinases catalytic subunit α (PIK3CA), Kirsten rat sarcoma viral oncogene homolog (KRAS), and epidermal growth factor receptor (EGFR), have been observed in cervical cancer patients.^4^ Recently, the chromobox protein homolog 7 (CBX7), which belongs to the polycomb group family, has been reported to regulate pluripotency of adult human pluripotent-like olfactory stem cells.^5^ In addition, one study showed that CBX7 regulates intrinsic axon growth and regeneration.^6^ CBX7 is identified to be lost in human malignant neoplasias.^7^ Moreover, downregulation of CBX7 is associated with poor prognosis and aggressiveness in human cancers.^7^ Furthermore, CBX7 regulates several genes that are critical for cancer development and progression, such as epithelial-mesenchymal transition (EMT) and drug resistance.^8^, ^9^ However, the biological function and role of CBX7 in cervical cancer have not been investigated, which is required to determine the CBX7 role in cervical progression.

In the current study, we investigated whether CBX7 exerts its tumor-suppressive function in cervical cancer cells. We used molecular approaches to upregulate the expression of CBX7 or downregulation of CBX7 in cervical cancer cell lines. Moreover, cell growth and apoptosis were measured in cervical cancer cells after CBX7 overexpression or downregulation. Furthermore, cell migration and invasion were determined in cervical cancer cells after CBX7 modulation. Mechanistically, E-cadherin and p65 expressions were measured by western blotting in cervical cells after CBX7 dysregulation. Our study will identify the role of CBX7 in cervical cancer.

## Results

### Overexpression of CBX7 Inhibits p65 and Induces E-cadherin Expression

**Figure 1:**
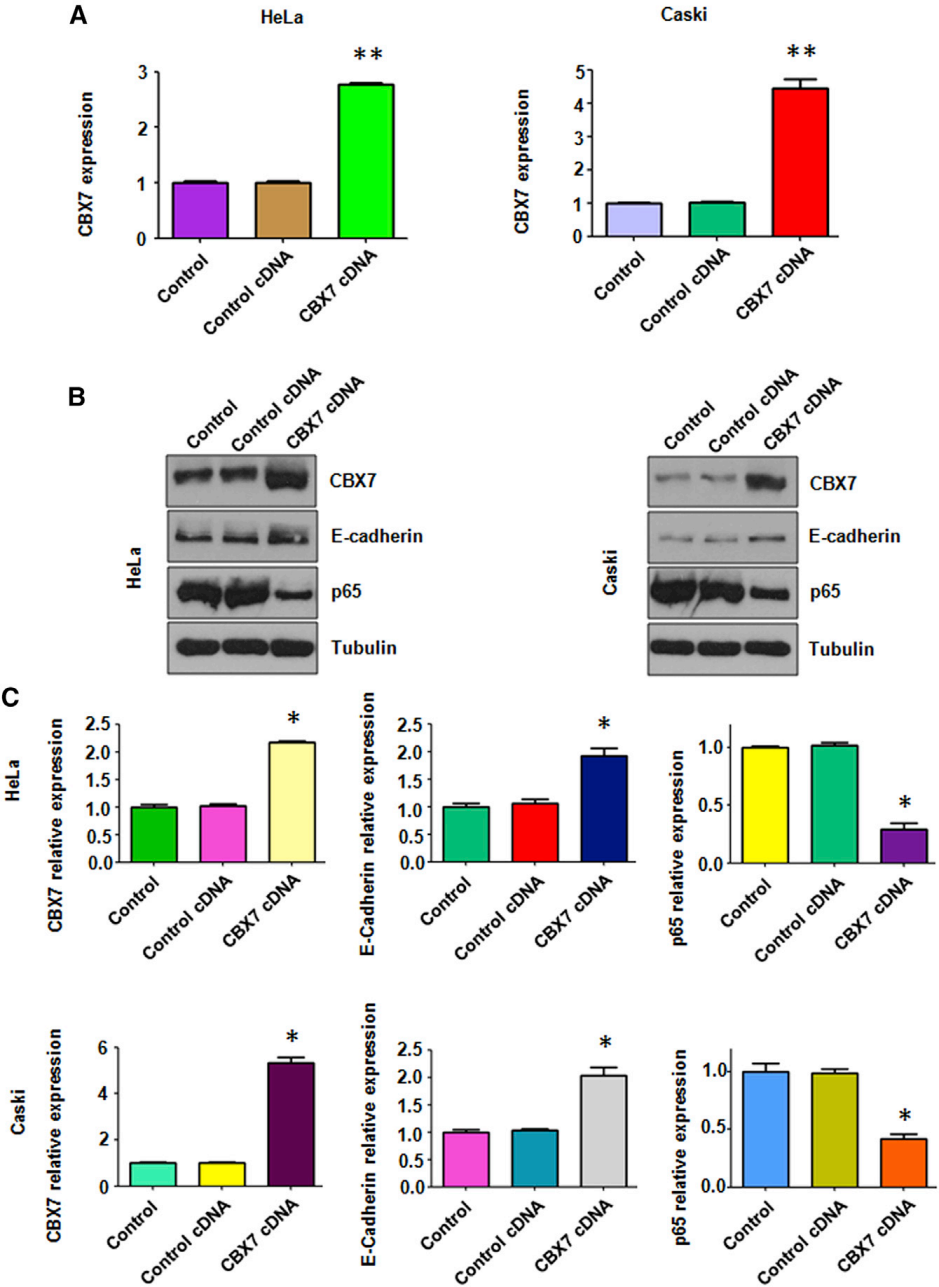
Overexpression of CBX7 Increases E-cadherin and Decreases the NF-κB Level (A) The CBX7 mRNA level was detected by real-time PCR in both HeLa and Caski cells with CBX7 construct transfection. **p < 0.01 versus control group. (B) Western blot was performed to analyze the protein level of CBX7, E-cadherin, and NF-κB in cervical cancer cells with CBX7 construct transfection. (C) Quantification of the expression of CBX7, E-cadherin, and NF-κB was performed. *p < 0.05 versus control group.

To investigate whether CBX7 plays an essential role in cervical cancer progression, cervical cancer cells were transfected with CBX7 cDNA vector or empty control. The mRNA level of CBX7 was measured by real-time RT-PCR analysis in cervical cancer cells after CBX7 cDNA transfection. Our RT-PCR results clearly showed that CBX7 mRNA level was significantly increased in cervical cancer cells after CBX7 cDNA transfection (Figure 1A). To test whether the protein levels of CBX7 was upregulated in cervical cancer cells after CBX7 cDNA transfection, western blotting analysis was used to measure the level of CBX7 expression. We found that the expression level of CBX7 was remarkably increased in both HeLa cells and Caski cells (Figures 1B and 1C). To determine further whether CBX7 overexpression was created in cells, we measured the downstream targets of CBX7, E-cadherin, and p65.^10^, ^11^ We found that overexpression of CBX7 increased the expression of E-cadherin but decreased the level of p65 in cervical cancer cells (Figures 1B and 1C). Taken together, CBX7 could regulate the expression of E-cadherin and the nuclear factor-kappa B (NF-κB) pathway in cervical cancer cells.

### Overexpression of CBX7 Inhibits Cell Proliferation

**Figure 2:**
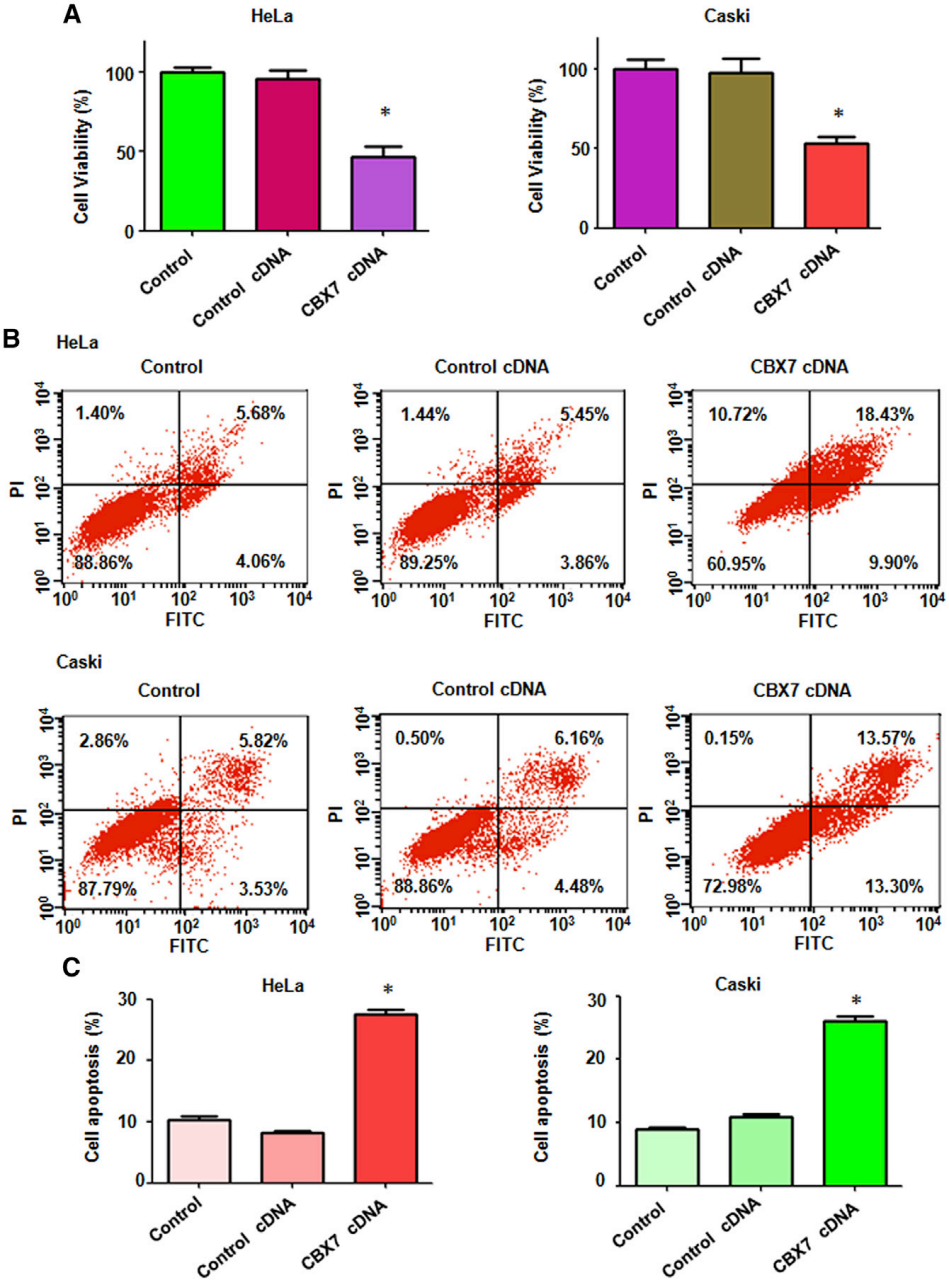
Overexpression of CBX7 Inhibits Cell Proliferation and Induces Apoptosis (A) Cell viability was measured by MTT in both HeLa and Caski cells with CBX7 construct transfection at 72 h. *p < 0.05 versus control group. (B) Cell apoptosis was determined by flow cytometry in cervical cancer cells with CBX7 construct transfection at 72 h. (C) Quantitative results are illustrated for cell apoptosis.

To determine whether overexpression of CBX7 governs cell proliferation in cervical cancer cells, the 3-(4,5-dimethylthiazol −2-yl)-2,5-diphenyltetrazolium bromide (MTT) assay was used to detect the proliferation of HeLa and Caski cells after CBX7 overexpression. We found that overexpression of CBX7 inhibited cell proliferation in both cervical cancer cell lines (Figure 2A). Specifically, CBX7 cDNA transfection led to a 50% reduction of cell proliferation (Figure 2A). These data clearly demonstrate that CBX7 inhibits cell proliferation, suggesting that CBX7 could play a tumor-suppressive role in cervical cancer.

### Overexpression of CBX7 Induces Cell Apoptosis

To explore the biological function of CBX7 in cervical cancer cells, apoptotic death was measured in HeLa cells and Caski cells after CBX7 overexpression. To achieve this goal, the propidium iodide (PI)-fluorescein isothiocyanate (FITC)-annexin assay was used to detect the cell apoptosis in both cervical cancer cells transfected with CBX7 cDNA. The cell apoptotic death of both cervical cancer cell lines was induced by CBX7 overexpression. The percentage of apoptosis was increased from 9.31% in the control cDNA group to 28.33% in the CBX7 cDNA transfection group in HeLa cells (Figures 2B and 2C). Likewise, the cell apoptosis was triggered from 10.64% to 26.87% after CBX7 overexpression in Caski cells (Figures 2B and 2C). This finding reveals that overexpression of CBX7 induces cell apoptosis in cervical cancer, which could contribute to cell growth inhibition.

### Overexpression of CBX7 Suppresses Cell Migration and Invasion

**Figure 3:**
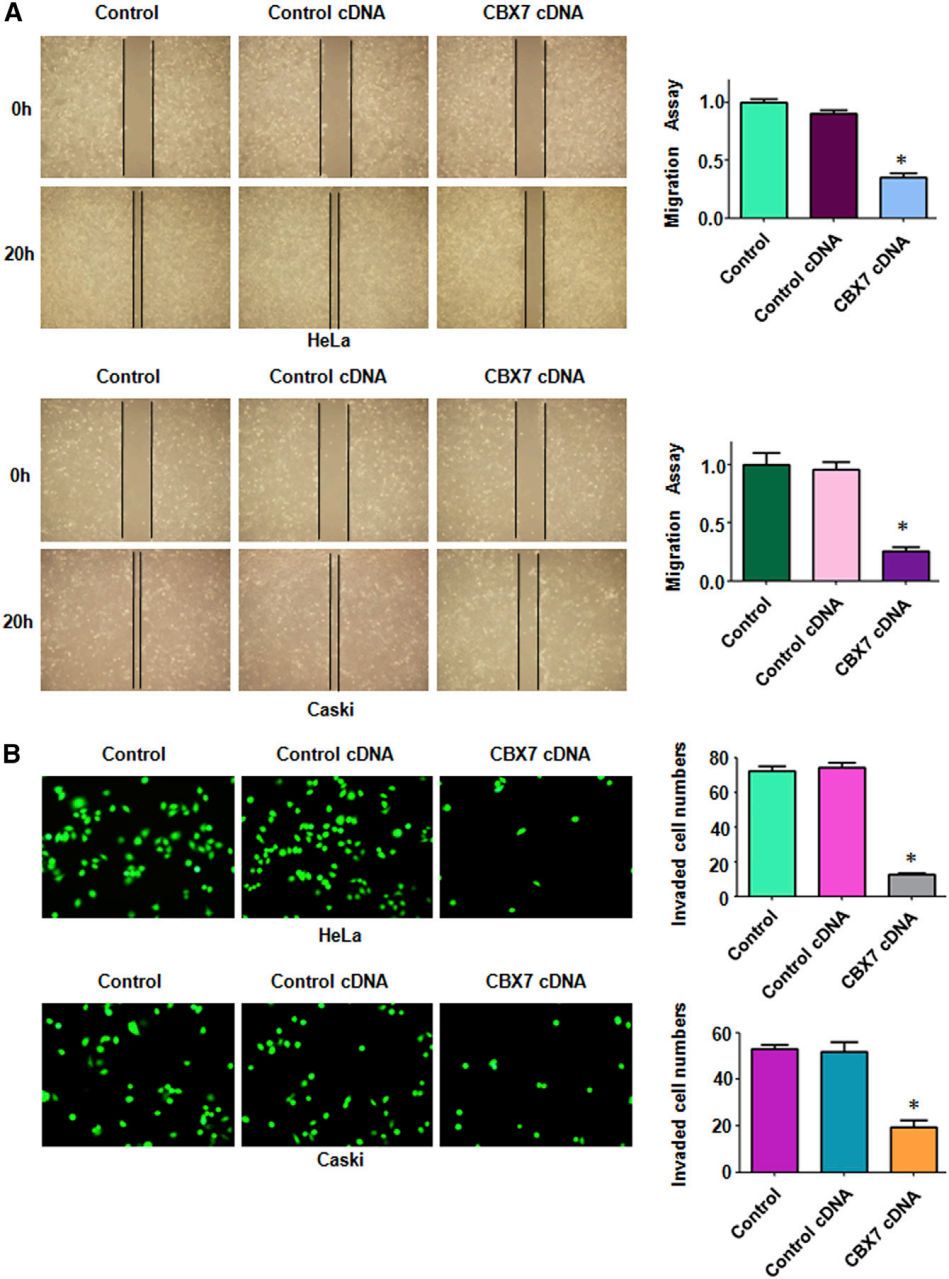
Overexpression of CBX7 Inhibits Cell Migration and Invasion (A) Left: the ability of cell migration was determined via a wound-healing assay in cervical cancer cells after CBX7 construct transfection. Right: Quantitative results are illustrated for left. *p < 0.05 versus control group. (B) Left: the ability of cell invasion was measured via the Transwell assay in cervical cancer cells after CBX7 construct transfection. Right: quantitative results are illustrated for left. *p < 0.05 versus control group.

CBX7 has been reported to inhibit the cell migration and invasion in several types of cancers.^8^, ^12^ However, it is unclear whether CBX7 regulates cell migration and invasion in cervical cancer. Therefore, we used a wound-healing assay to measure the migration in cervical cancer cells after CBX7 overexpression for 20 h. We found that overexpression of CBX7 inhibited the migration of HeLa cells and Caski cells (Figure 3A). Moreover, a Transwell chamber invasion assay was performed to examine the invasive activity of cervical cancer cells after CBX7 overexpression for 20 h. The invasion result showed that CBX7 overexpression suppressed cell invasion in HeLa cells and Caski cells (Figure 3B). Because CBX7 overexpression did not change the cell growth at 24 h (data not shown), CBX7 overexpression retarded cell motility in cervical cancer cells, not because of cell growth inhibition by CBX7 upregulation.

### Downregulation of CBX7 Inhibits E-cadherin and Increases NF-κB

**Figure 4:**
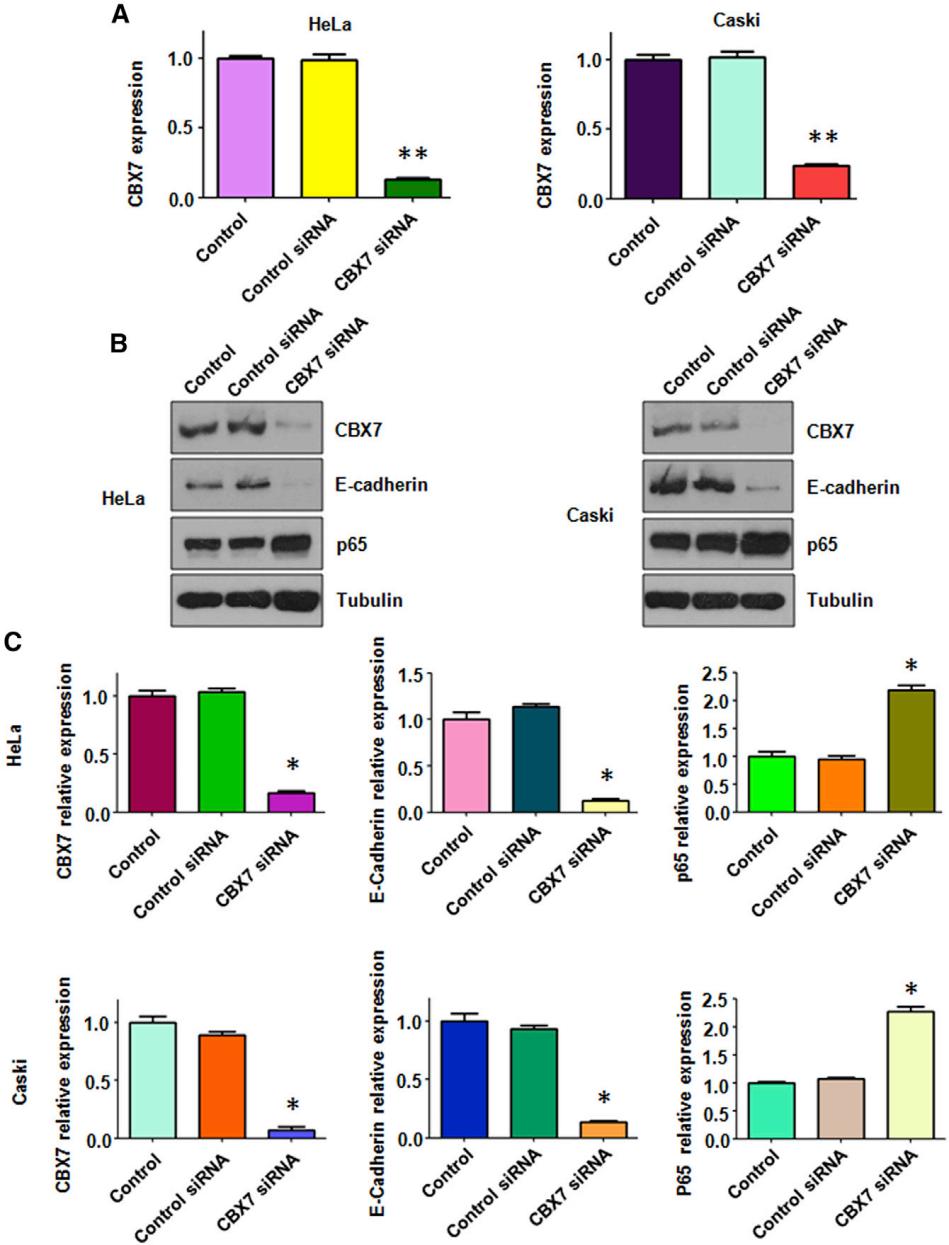
Downregulation of CBX7 Decreases E-cadherin and Increases the NF-κB Level (A) The CBX7 mRNA level was detected by real-time PCR in both HeLa and Caski cells with CBX7 siRNA transfection.**p < 0.01 versus control group. (B) Western blot was performed to analyze the protein level of CBX7, E-cadherin, and NF-κB in cervical cancer cells with CBX7 siRNA transfection. (C) Quantification of the expression of CBX7, E-cadherin, and NF-κB was performed. *p < 0.05 versus control group.

To investigate deeper the role of CBX7 in cervical cancer cells, CBX7 small interfering RNA (siRNA) was used to downregulate the expression of CBX7. The efficacy of CBX7 siRNA was determined by RT-PCR and western blotting analysis, respectively. Our RT-PCR results showed that CBX7 siRNA transfection decreased the mRNA level of CBX7 in cervical cancer cells (Figure 4A). Consistently, the results from our western blotting revealed that CBX7 siRNA transfection downregulated the expression of the CBX7 protein in both cervical cancer cell lines (Figures 4B and 4C). Notably, we found that CBX7 downregulation inhibited the expression of E-cadherin and increased the NF-κB level in HeLa and Caski cells (Figures 4B and 4C).

### Downregulation of CBX7 Promotes Cell Proliferation

**Figure 5:**
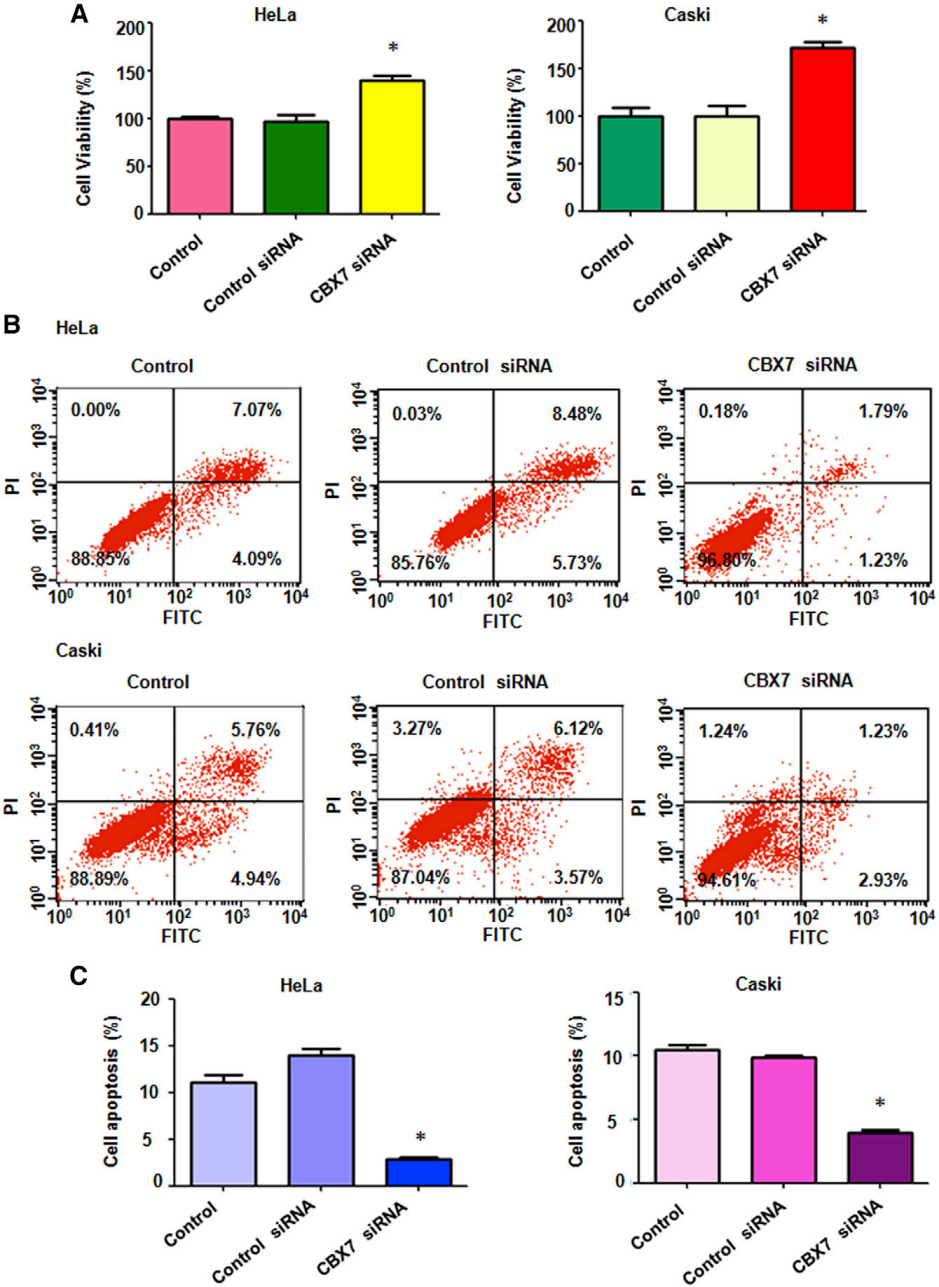
Downregulation of CBX7 Promotes Cell Proliferation and Inhibits Apoptosis (A) Cell viability was measured by MTT in both HeLa and Caski cells with CBX7 siRNA transfection at 72 h. *p < 0.05 versus control group. (B) Cell apoptosis was determined by flow cytometry in cervical cancer cells with CBX7 siRNA transfection at 72 h. (C) Quantitative results are illustrated for cell apoptosis.

Next, we examined cell proliferation in cervical cancer cells after CBX7 downregulation by the MTT assay. We found that CBX7 downregulation promoted cell proliferation in both cervical cancer cell lines (Figure 5A). Downregulation of CBX7 in HeLa cells led to 40% of cell proliferation promotion (Figure 5A). Likewise, CBX7 downregulation in Caski cells resulted in an increased 80% of cell proliferation (Figure 5A). These data clearly demonstrate that CBX7 governs cell proliferation in cervical cancer.

### Downregulation of CBX7 Inhibits Cell Apoptosis

The Annexin V-FITC/PI apoptosis detection kit was applied to examine the cell apoptosis in cervical cancer cells after CBX7 siRNA transfection. We observed that CBX7 downregulation inhibited cell apoptosis in both cervical cancer cells (Figures 5B and 5C). The percentage of apoptotic death cells was decreased from 14.21% in the control siRNA treatment group to 3.02% in the CBX7 siRNA treatment group in HeLa cells (Figure 5B). Likewise, the percentage of cell apoptosis was decreased from 9.69% to 4.16% in Caski cells after CBX7 downregulation (Figure 5B). Our results indicate that CBX7 regulates the cell apoptosis in cervical cancer.

### Downregulation of CBX7 Promotes Cell Migration and Invasion

**Figure 6:**
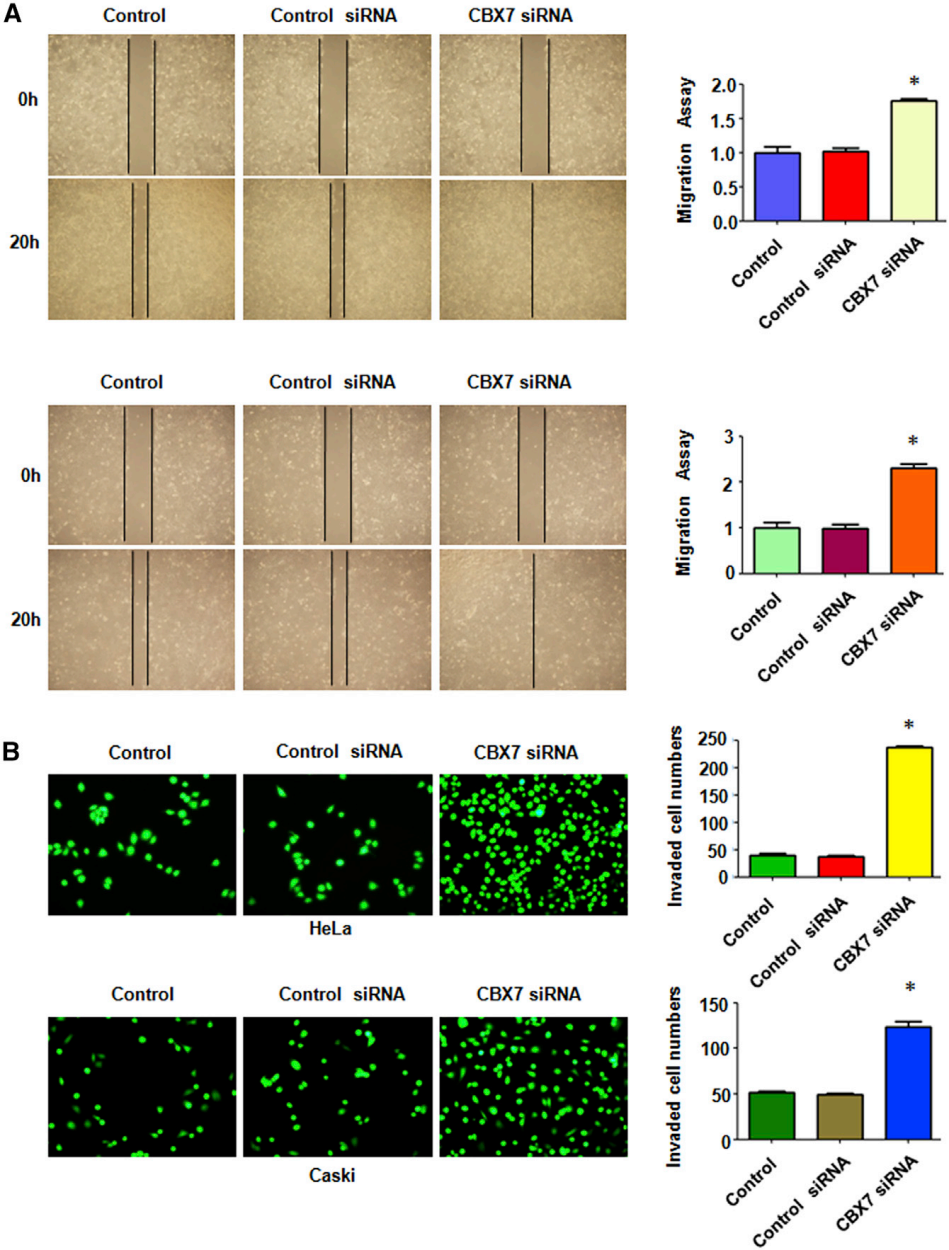
Downregulation of CBX7 Promotes Cell Migration and Invasion (A) Left: The ability of cell migration was determined via a wound-healing assay in cervical cancer cells after CBX7 siRNA transfection. Right: Quantitative results are illustrated for left. *p < 0.05 versus control group. (B) Left: the ability of cell invasion was measured via Transwell assay in cervical cancer cells after CBX7 siRNA transfection. Right: quantitative results are illustrated for left. *p < 0.05 versus control group.

To investigate the effect of CBX7 downregulation on cell migration, a wound-healing assay was conducted in cervical cancer cells after CBX7 siRNA transfection for 20 h. The results from the wound-healing assay showed that CBX7 downregulation enhanced cell migration in both cervical cancer cell lines (Figure 6A). Furthermore, our Transwell chamber assay data demonstrated that CBX7 downregulation promoted cell invasion in HeLa and Caski cells (Figure 6B). As CBX7 siRNA transfection did not change the cell growth at 24 h (data not shown), the enhancement of cell motility induced by CBX7 downregulation is not a result of cell growth change by CBX7 modulation. These findings clearly suggest that CBX7 downregulation enhances cell motility in cervical cancer cells.

## Discussion

CBX7 is initially reported to extend the lifespan of a wide range of normal human cells through downregulation of Ink4a/Arf locus expression.^13^ Recently, evidence has demonstrated that CBX7 is involved in tumorigenesis. Loss of CBX7 expression is associated with malignancy grade in human pancreatic cancer, whereas high expression of CBX7 in patients has a longer survival.^14^ Likewise, loss of the CBX7 expression is correlated with a highly malignant phenotype in thyroid cancer.^15^ Moreover, CBX7 is downregulated in colon cancer and is associated with lymph metastasis and poor overall survival in patients with colon cancer.^16^ Another study showed that CBX7 is a prognostic biomarker in glioma patients and induces cell-cycle arrest through downregulation of cyclin E.^17^ Furthermore, expression of CBX7 is correlated with poor prognosis in ovarian clear cell adenocarcinoma patients, thyroid cancer.^15^, ^18^ In line with these reports, CBX7 overexpression is associated with better relapse-free survival of breast cancer patients.^19^ Single nucleotide polymorphisms of CBX7 reduce the risk of hepatocellular carcinoma (HCC).^20^ Downregulation of CBX7 is associated with HCC progression and poor prognosis in patients with HCC.^21^ Moreover, CBX7 was found to induce self-renewal of malignant hematopoietic stem and progenitor cells.^22^ However, several studies have shown that CBX7 could be an oncogene in multiple types of human cancers. For example, higher expression of CBX7 was correlated with clinical stage and lymph node metastasis in gastric cancer.^13^ Downregulation of CBX7 inhibited cellular proliferation and migration ability via increased p16 in gastric cancer.^13^ Notably, CBX7 positively regulates stem cell-like characteristics through activation of the Akt-NF-κB pathway and inhibition of p16 in gastric cancer.^11^ The function of CBX7 in cervical cancer has not been elucidated. Herein, we report that CBX7 plays a tumor-suppressive role in cervical cancer.

Accumulated evidence has dissected the mechanism of CBX7 in regulation of cell growth in human cancer. For instance, CBX7 governs the growth of prostate cancer cells via repression of the Ink4a/Arf locus.^23^ Moreover, restoration of CBX7 expression inhibits cell growth via retention of the cell cycle in thyroid cancer cells.^15^ One study indicates that CBX7 suppresses cell proliferation through the suppression of the Akt signaling pathway in pancreatic cancer.^12^ In line with this, our study also showed that CBX7 overexpression inhibited cell growth and induced apoptosis in cervical cancer cells. Several studies have shown that CBX7 could regulate the expression of E-cadherin in a variety of human cancers. For example, CBX7 was reported to regulate E-cadherin expression positively through interaction with the histone deacetylase 2 (HDAC2) protein.^10^ CBX7 overexpression upregulated E-cadherin expression and increased the acetylation status of the histones H3 and H4 on the promoter of E-cadherin. Moreover, CBX7 expression is positively correlated with the E-cadherin level in human thyroid carcinomas.^10^ Likewise, loss of CBX7 is correlated with loss of E-cadherin and with a worse survival in pancreatic cancer patients.^14^ One study showed that augmentation of CBX7 by miR-182 knockdown positively regulated E-cadherin expression in human breast cancer.^24^ In addition, CBX7 was found to regulate negatively migration and invasion via upregulation of E-cadherin and downregulation of matrix metalloproteinase (MMP)-2, MMP-9, and vimentin in glioma.^8^ Recently, one group revealed that CBX7 increased E-cadherin expression via interaction with protein arginine methyltransferase 1 (PRMT1) and HDAC2.^25^ In line with these reports, we also observed that CBX7 positively regulated the expression of E-cadherin in cervical cancer cells, which could be the reason for inhibition of cell migration and invasion.

One study has demonstrated that restoration of CBX7 expression increases the susceptibility to irinotecan treatment in human lung carcinoma cells.^9^ Recently, a number of microRNAs (miRNAs) have been identified to regulate the expression of CBX7 in human cancers. One group reports that miR-421 decreases cell growth via upregulation of CBX7 in gastric cancer cells.^26^ Moreover, miR-181b negatively regulates CBX7 expression in breast cancer, leading to promotion of cell-cycle progression.^27^ Augmentation of CBX7 via knockdown of miR-182 expression governs cell morphology via the expression of E-cadherin in breast cancer.^24^ Two studies showed that miR-9 targets the expression of CBX7 in bladder cancer cells.^28^, ^29^ Furthermore, CBX7 is found to be a target of miR-375 in prostate cancer progression.^30^ Likewise, miR-18a plays a malignant role via directly targeting CBX7 in human glioblastoma.^31^ Strikingly, miR-19 enhances cell proliferation via inhibition of CBX7 expression in nonsmall cell lung cancer cells.^32^ These findings suggest that restoration of CBX7 could be via inhibition of these miRNAs that target CBX7 in human cancers. Taken together, CBX7 could be a potential target in cervical cancer.

## Materials and Methods

### Cell Culture and Agents

The HeLa and Caski cells were cultured in RPMI-1640 medium supplemented with 10% fetal bovine serum (FBS) and 1% penicillin and streptomycin at 37°C in 5% CO_2_ atmosphere. The anti-E-cadherin antibody (#ab15148) and anti-CBX7 antibody (#ab91431) were purchased from Abcam. The antibody against p65 (#4764) was purchased from Cell Signaling Technology (Danvers, MA, USA). Anti-tubulin antibody (#SC5286) was bought from Santa Cruz Biotechnology (Santa Cruz, CA). The second antibodies were purchased from Thermo Scientific. Lipofectamine 3000 was purchased from Invitrogen.

### Transfection

The cervical cancer cells were transfected with control cDNA or CBX7 cDNA or control siRNA or CBX7 siRNAs (Genepharma, Shanghai, China) via Lipofectamine 3000, following the instruction’s protocol.^33^ The sequence of CBX7 siRNA was as follows: 5′-CAC CTT GCA TGC ACC TTG CTA-3′. Then, the transfected cells were seeded into 96-well or six-well plates to analyze further, as described in Results.

### Cell Growth Assay

The cervical cancer cells were seeded into 96-well culture plates. After overnight incubation, cells were transfected with CBX7 siRNA or CBX7 cDNA for 72 h. The MTT assay was performed as described previously.^34^

### Cell Apoptosis Assay

The cervical cancer cells were transfected with CBX7 cDNA or CBX7 siRNAs via Lipofectamine 3000. The transfected cells were seeded in six-well plates for 72 h. Then the cells were collected and washed once with PBS and suspended in binding buffer, including 5 μL PI and 5 μL FITC-conjugated anti-Annexin V antibody. Apoptotic cell numbers were measured by a FACSCalibur flow cytometer (BD, USA), as described previously.^35^

### Wound-Healing Assay

The transfected cervical cancer cells were seeded in six-well plates and waited for almost >90% confluency. Then, wound was created by a small yellow pipette tip and washed with PBS. The wound area was photographed with a microscope at 0 h and 20 h, respectively.^34^

### Cell-Invasion Assay

The transfected cervical cancer cells were seeded onto the upper chamber with the Matrigel-coated membrane in 200 μL serum-free medium. The lower chamber was added with RPMI medium containing 10% FBS. After 20 h, the invaded cells were stained with 4 μg/mL calcein-AM at 37°C for 1 h. Invasiveness was imaged by a microscope.^34^

### Quantitative Real-Time RT-PCR Analysis

Total RNA was extracted from the transfected cervical cancer cells. Then, the cDNA was generated by reverse transcription (RT) using oligo (dT) primers. PCR was performed using Power SYBR Green PCR Master Mix, as described previously.^33^ The primers used in the PCR reaction are as follows: CBX7, forward primer (5′-CAT GGA GCT GTC AGC CAT C-3′) and reverse primer (5′-CTG TAC TTT GGG GGC CAT C-3′);^36^ GAPDH, forward primer (5′-ACC CAG AAG ACT GTG GAT GG-3′) and reverse primer (5′-CAG TGA GCT TCC CGT TCA G-3′).

### Western Blotting Analysis

The transfected cervical cancer cells were lysed in lysis buffer. Then, proteins in cell lysis were extracted and loaded into each lane in equal amounts and resolved by sodium dodecyl sulfate-polyacrylamide gel electrophoresis (SDS-PAGE), transferred to nitrocellulose membranes. Then, the membranes were incubated with the primary antibodies at 4°C overnight, including anti-CBX7 (1:1,000), anti-p65 (1:2,000), anti-E-cadherin (1:1,000), and anti-tubulin (1:3,000) antibodies. Then, the membranes were incubated with secondary antibodies conjugated with horseradish peroxidase. Lastly, bands were measured using an enzyme-linked chemiluminescence detection kit (ECL) assay.^34^

### Statistical Analysis

All statistical data were conducted by GraphPad Prism 5.0 (Graph Pad, La Jolla, CA). Analysis of variance (ANOVA) was used to evaluate significance among different groups. The results were presented as means ± SD. p < 0.05 was considered statistically significant.

## Author Contributions

R.L., Q.Y., and P.T. performed the experiments. Y.W., J.W., N.T., L.N., and X.L. analyzed the data. R.L. and L.D. wrote the manuscript. L.D., J.L., and C.M. critically viewed and supervised the study.

## Conflicts of Interest

The authors declare no competing interests.
